# Tumor Necrosis Factor Receptor 2: Its Contribution to Acute Cellular Rejection and Clear Cell Renal Carcinoma

**DOI:** 10.1155/2013/821310

**Published:** 2013-11-17

**Authors:** Jun Wang, Rafia S. Al-Lamki

**Affiliations:** ^1^Department of Nephrology, First Hospital of China Medical University, Shenyang, China; ^2^Department of Medicine, Cambridge University NHS Trust Hospitals, Addenbrooke's Hospital, P.O. Box 157, Level 5, Hill's Road, Cambridge CB2 0QQ, UK

## Abstract

Tumor necrosis factor receptor 2 (TNFR2) is a type I transmembrane glycoprotein and one of the two receptors that orchestrate the complex biological functions of tumor necrosis factor (TNF, also designed TNF-**α**). Accumulating experimental evidence suggests that TNFR2 plays an important role in renal disorders associated with acute cellular rejection and clear cell renal carcinoma but its exact role in these settings is still not completely understood. This papers reviews the factors that may mediate TNFR2 induction in acute cellular rejection and clear cell renal carcinoma and its contribution to these conditions and discusses its therapeutic implications. A greater understanding of the function of TNFR2 may lead to the development of new anti-TNF drugs.

## 1. Introduction

Tumor necrosis factor (TNF, also known as TNF-*α*) is a type II transmembrane protein with an intracellular aminoterminus. It can transduce signalling both as a membrane-integrated protein and as a soluble cytokine released after proteolytic cleavage; its soluble form is a noncovalently bound trimer of 17 kDa components [1] and its transmembrane form is a functional 26-kDa homotrimer protein [2]. TNF is a major mediator of inflammatory, immunological, and pathophysiological reactions [3]. TNF mediates various cellular responses by binding to two structurally distinct receptors: TNF receptor 1 (TNFR1; TNFRSF1A, CD120a, and p55) and TNF receptor 2 (TNFR2; TNFRSF1B, CD120b, and p75), members of the TNFR superfamily [4, 5]. TNFRs share homology in their extracellular regions, containing cysteine-rich structural motifs that form TNF-binding domains, but have nonidentical intracellular domains resulting in nonoverlapping functions. The affinities of both receptors for soluble TNF are similar. In contrast to TNFR1, which belongs to the subgroup of TNFR superfamily molecules that contain a cytoplasmic death domain (DD), TNFR2 does not have a DD and lacks the ability to directly activate the apoptotic machinery through DD adaptors [6]. Compared to TNFR1, which is detected on nearly all kinds of cells and predominantly sequestered in the Golgi apparatus [7], TNFR2 exhibits far more limited expression and is typically localized to the plasma membrane in epithelial cells of the distal collecting tubules in acute transplant rejection [8], in T-lymphocytes [9], cardiomyocytes [10], human mesenchymal stem cells [11], human and murine cardiac resident stem cells [12], microglia and specific neuron subtypes [13], oligodendrocytes [14], and thymocytes [15]. Most biological responses have been attributed to signalling initiated by TNFR1, but some responses have been ascribed to ligand binding to TNFR2 [10, 16, 17]. Signal transduction through TNFR1 has been widely studied over the past few years, whereas TNFR2 has proved to be far more elusive in revealing its signal transduction pathways, although recent evidence suggests that it plays an essential role in cell proliferation and survival [9–11, 18, 19] and in the activation of regulatory T cells [20, 21].

Kidney transplantation is the best choice for the treatment of end-stage kidney disease but rejection is the major complication after transplant resulting in graft loss. Acute cellular rejection (ACR) involves the interplay between mechanisms that maintain tolerance to the graft and factors that promote rejection [22]. Cytokines are potent mediators of alloimmune response leading to ACR. Increased levels of TNF mRNA are expressed in ACR and TNF protein is secreted primarily by immune cells such as macrophages, lymphocytes, natural killer cells, and nonlymphoid cells, for example, endothelial cells (ECs) and tubular epithelial cells (TECs), and are detected in rejecting kidney grafts [10, 23]. However, the roles of TNFRs in the setting of ACR are yet still unclear.

Renal cell carcinoma (RCC), a TEC malignancy, is the most common type of kidney cancer in adults, responsible for approximately 80% of cases [24]. A clear cell renal carcinoma (ccRCC) is the major subtype of RCC accounting for >80% of all RCC types, while the other 20% comprise of papillary, chromophobe, and oncocytic types [25]. RCC is relatively resistant to conventional cancer treatments, such as radio-, immuno-, and chemotherapy [26]. Although surgery and cryoablation are successful curative treatments for localized RCC, most patients are diagnosed with advanced or metastatic RCC with poor prognosis. The aggressive nature of RCC is reflected by recurrence rates of 20% to 40% after nephrectomy [27]. The risk of renal transplantation patients developing *de novo* malignancy is 100-fold increase compared with the healthy nontransplantation population [28–30]. Up to 4.6% of the renal transplant recipients are diagnosed with RCC arising from allografted kidney as a consequence of immunosuppression [29, 30]. Current staging systems provide good prognostic information, however, identification of new more accurate and predictive prognostic markers, not currently included in traditional staging systems, will improve the outcome for RCC patients. For this reason, increased knowledge of the underlying molecular characteristics of RCC development and progression is necessary. The expression and anatomical distribution of the TNFRs is certainly of great importance in trying to understand the pathophysiology of TNF responses. TNFRs are differentially expressed in normal kidney and their expression is modulated during immune-mediated renal injury [8, 18] and in ccRCC [16], with TNFR2 significantly induced in injured and malignant TECs. In this review, we will discuss the contribution of TNFR2 in ACR and ccRCC and its therapeutic implications.

## 2. TNFR2 Signalling and Biological Responses

**Figure 1:**
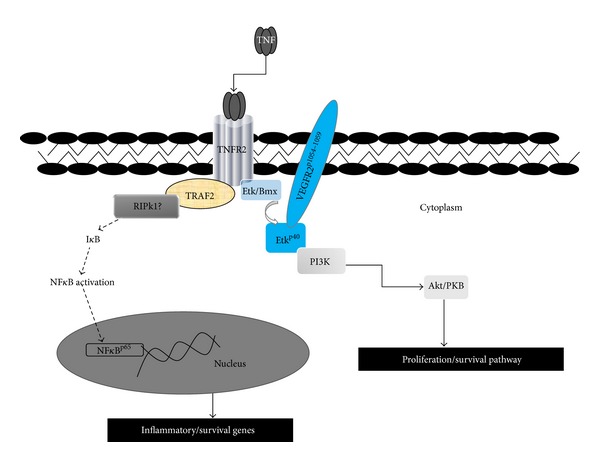
Intracellular tumour necrosis factor receptor-2 (TNFR2) signalling. Binding of tumour necrosis factor (TNF) to TNFR2 results in activation and recruitment of intracellular adaptor proteins that induce signal transduction promoting cell proliferation and survival via phosphorylation of endothelial/epithelial nonreceptor tyrosine kinase (Etk^p40^), which in turn transactivates vascular endothelial growth factor receptor-2 (VEGFR2^p1054–1059^), leading to phosphatidylinositol 3-kinase (PI3K)-Akt/PKB pathway.

TNFR2 signaling pathways are illustrated in Figure 1. TNFR2 was initially believed to display modulating and supportive functions to TNFR1, a process called “*ligand passing*” [31]. However, further characterization of the TNFRs using TNFR-specific muteins (mutant forms of TNF that engage exclusively to TNFR1 or TNFR2 so are receptor-specific ligands) or agonistic antibodies have led to the elucidation of different signaling pathways engaged by the two TNFRs. TNFR2 is a main receptor for transmembrane TNF, which binds more strongly to TNFR2 than does soluble TNF due to the formation of a tight trimer that is unable to dissociate from the receptor [32].

Transduction of extracellular signals by TNFR2 occurs via recruitment of cytosolic proteins to its intracellular domain as a result of the conformational changes induced by ligand binding [32]. The ligand-bound TNFR2 initiates signalling via direct binding to TNFR-associated factor 2 (TRAF2) [33]. TRAF1 and TRAF2 are associated with the cytoplasmic domain of TNFR2 as a heterodimeric complex in which only TRAF2 contacts the receptor directly. TNFR2 can also recruit TRAF2 and activate the two cellular inhibitors of apoptotic proteins (cIAP1 and cIAP2) [34]. The ubiquitin protein ligase activity of c-IAP1 is responsible for regulating the duration of TNF signaling in primary cells expressing TNFR2 [35]; however, the exact role of these factors in TNFR2-specific signalling remains undefined. The TNFR2/TRAF2 complex provides a mechanism for activation of transcription factors such as nuclear factor-*κ*B (NF*κ*B) and the activator protein 1 (AP-1) [36]. NF*κ*B is a critical transcription factor that plays an important role in cell growth, survival, and immune regulation. AP-1 is a heterodimeric transcription factor, the most common form consisting of c-Jun and c-Fos [37]. However, TNFR2 expressed at physiological levels fail to induce significant NF*κ*B and c-Jun N-terminal kinase (JNK) activation [38]. Only when over-expressed, TNFR2 can display NF*κ*B and JNK activation by TNF treatment [39]. Thus, it appears that TNFR2 is a weak trigger of NF*κ*B and JNK activation despite its high TRAF2 binding capability. This may be attributed to a novel carboxyl-terminal TRAF2-binding site (T2bs-C) that prevents the delivery of activation signals from the conventional TRAF2 binding site (T2bs-N) [40]. However, others have reported that the C-terminal region of TNFR2 responsible for the binding of TRAF2 is indispensable for signal transduction and NF*κ*B activation [41]. Another mechanism by which TRAF2 can activate NF*κ*B involves RIPK1 [42]. Indeed, *rip*−/− mice (lacking RIPK1) show no NF*κ*B activation in response to TNF, and TNFR2 was identified as the primary receptor that induced I*κ*B*α* degradation, the initiating event in NF*κ*B activation [43]. In keeping with this observation, neutralization of TNFR2 on podocytes with blocking antibodies abrogated TNF-induced NF*κ*B activation and induced cyclin D1 instead [44]. Regardless of the mechanism, the key point is that the activation of NF*κ*B cannot be exclusively relied on as a marker for signalling via TNFR2 as ligand-bound TNFR2 can activate NF*κ*B in only some cell types. Following ligation of TNFR2, the downstream signaling effects can depend on which of the genes NF*κ*B transcribes. Although the effect is most frequently proliferation, it can include differentiation, cytokine production and apoptosis. TNFR2 activation can induce cell death in T lymphocytes [42]; however, the signalling pathway responsible for this is still unclear although TNF can also kill other cell types via TNFR2 activation when phosphatidylinositol 3 kinase (PI3K) is blocked [45], probably through necroptosis [46]. TNFR2 can promote proliferation/survival in renal tubular cells, renal tumor cells, and ECs via phosphorylation of a cytosolic protein tyrosine kinase, belonging to the btk family, also known as epithelial/endothelial tyrosine kinase (Etk) in humans and as bone marrow X-linked kinase (Bmx) in mice [10, 16, 18, 47]. TNFR2-induced activation of Etk causes transactivation of vascular endothelial growth factor receptor-2 (VEGFR2, also known as KDR or flk-1) [16, 47] resulting in activation of PI3K and its effector Akt, a serine/threonine kinase also called protein kinase B or PKB, leading to cell proliferation/survival. Murine heart lacking TNFR2 shows a diminished activation of Etk [10]. Thus, phosphorylation of Etk may be useful to assess TNFR2 responses *in situ*, where downstream signalling has been shown to mediate reno- and cardioprotection [10, 18].

## 3. TNFR2 and ACR

**Figure 2:**
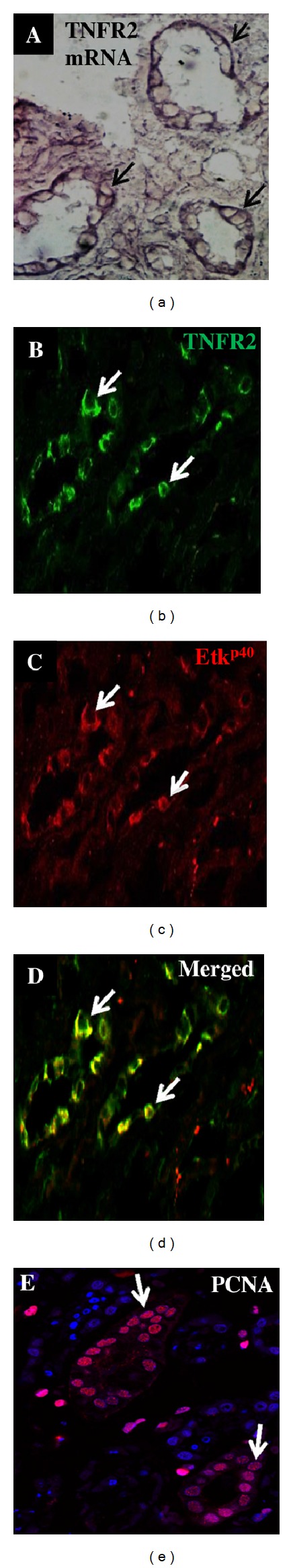
Tumour necrosis factor receptor-2 (TNFR2) mediated cellular responses in organ cultures of ACR. Treatment of cultures with a TNFR2-selective mutein induces TNFR2 mRNA and protein in tubular epithelial cells (arrows) (a, b), which in turn activates and phosphorylates epithelial/endothelial tyrosine kinase at tyrosine 40 (Etk^p40^) (c, d) promoting cell cycle entry, indicated by staining for proliferative cell nuclear antigen (PCNA) (e).

TNF is a mediator of tissue injury associated with allograft rejection after renal transplantation [48]. TNF gene polymorphism at position G/A-308 in donors with high levels of TNF production is associated with a higher risk of rejection, delayed graft function/survival in kidney transplant recipients [49, 50]. However, some reports fail to show an association [51] and conflicting data exist as to whether serum/urine TNF levels reflect reliably the rejection crisis [52]. The effect of TNF on graft outcome is influenced by the diverse range of cellular responses triggered by the two distinct TNFRs, which lead to signalling cascades that induce expression of many genes involved in inflammation. TNFRs are differentially expressed and regulated in normal human kidney and in renal transplants undergoing ACR [8]. In contrast to TNFR1, mainly present in glomerular and perivascular capillaries ECs and in infiltrating leukocytes in human renal allograft biopsies, TNFR2 is significantly induced in injured TECs near TNF-expressing leukocytes and in ECs in humans [8, 18] and rats [53] but barely detected in kidney of healthy subjects and in normal rats. TNFR2 inducibility is supported by its promoter region, which has a cAMP-response and consensus elements for a number of transcription factors including NF*κ*B, AP-1, IRF, and GAS [54]. Interaction of TNF and TNFR2 has been demonstrated in different models of immune-mediated renal injury [10, 18, 53, 55]. In organ culture of normal human kidney and allograft rejection, selective engagement of TNFR2 by a specific TNF mutein induces TNFR2 protein and mRNA in TECs (Figure 2), suggesting that the receptor is controlled at the level of gene transcription [8, 10, 18, 56]. TNFR2 mRNA has a large 3′ untranslated region, which, in addition, may be important in regulation of TNFR2 mRNA stability posttranscriptionally [57]. 

The induction of TNFR2 protein and mRNA in TECs in allograft rejection strongly suggests that it is an important receptor in mediating and/or regulating the rejection process. TNFR2 has been shown to participate in the process of xenograft rejection in pigs [58] and its soluble form acts as a powerful immunosuppressant in alloreactivity [59]. However, the factors that mediate induction of TNFR2 in injured TECs and its contribution to the process of rejection are incompletely understood. Based on our work in human organ cultures [10, 18, 56] and of other groups [44, 60], we can postulate several possibilities as to the roles of TNFR2 in ACR. The pathological changes in intragraft microenvironment during development of allograft rejection may promote/sustain TNFR2 expression in an autocrine and/or paracrine manner. EC destruction/proliferation, resulting from infiltration of alloimmune leukocyte- and/or alloantibody-induced responses, leading to abnormal blood flow and hypoxia in tissue [56, 61] may be important contributors to TNFR2 expression/function in TECs [10, 56]. The effect of hypoxia on TNFR2 regulation has recently been reported [12].

Renal TECs, the predominant parenchymal cell type in the kidney and the principal target of renal injury, can be induced to synthesize/secrete inflammatory mediators including TNF, interleukin (IL)-6 and -10, interferon-*γ* (IFN-*γ*), and chemokines which mediate activation and recruitment of leukocytes within the graft tissue as a hallmark of allograft rejection [62–64]. The invading immune cells including T- and B-lymphocytes, Tregs, and tissue-associated macrophages (TAM) proliferate and are fully activated *in situ* in tubulitis associated with ACR and may be instrumental in inducing TNFR2 in TECs via production of TNF [8, 18]. 

Another factor that may regulate TNFR2 induction in TECs is TNF-converting enzyme (TACE, also known as ADAM-17) synthesized *in situ* by TECs and colocalizes with TNFR2 during ACR [65]. TACE activity is regulated primarily by controlling its localization. Inactive rhomboid protein 2 (iRhom2), a proteolytically inactive member of the rhomboid family, binds TACE and promotes its exit from the endoplasmic reticulum [66], allowing it to traffic to the cell surface. TACE regulates shedding of TNFR2 into the local environment, which may attenuate inflammation caused by TNF [65]. Indeed, changes in serum levels of TNFR2 correlate significantly with renal transplant rejection [67] and renal injury in mice [44]. The soluble shed TNFR2, which is capable of binding TNF, may neutralize TNF-induced cytotoxicity and immune-reactivity thus downregulating inflammation *in vivo* and *in vitro* [60]. 

Another important mediator of TNFR2 induction in TECs is TL1A (TNFSF15; also known as VEGI), the principal ligand for DR3 [56, 68]. TL1A is secreted and released by vascular ECs and infiltrating leukocytes in acute and antibody-mediated allograft rejection [56]. TECs expresses TL1A protein but not its mRNA, consistent with its uptake as an exogenous ligand by TECs [56]. In kidney organ cultures from DR3-deficient mice, TL1A induces TNFR2 upregulation in TECs via NF*κ*B-independent pathways [56]. Hence, leukocyte/ECs-derived TL1A in allograft rejection may contribute to the induction of  TNFR2 in TECs *in situ*. 

TNFR2 protein and mRNA can also be induced in organ culture of renal allograft rejection treated with wild type TNF or a TNFR2-selective mutein [18], resulting in phosphorylation of Etk. TNFR2-mediated Etk phosphorylation leads to activation of the PI3K/Akt pathway providing a signal for tubular regeneration by promoting cell proliferation (indicated by expression of a cell cycle marker Proliferation Cell Nuclear Antigen; PCNA) (Figure 2) [18, 47]. Indeed, *in vivo* and *in vitro* studies using a TNFR2-specific mutein and TNFR2-deficient cells have confirmed that Etk is a TNFR2-specific kinase involved in cell adhesion, migration, and survival [47]. Collectively, current data implicate TNFR2 in ACR, albeit the causal connections remain to be revealed. 

## 4. TNFR2 and ccRCC 

RCC is typified by biallelic inactivation of the von Hippel-Lindau (VHL) tumor suppressor gene [69]. A mutation in VHL gene results in increased production of cytokines including TNF, which sensitizes local tumor cells to TNF actions. TNF is a major mediator of cancer-related inflammation [70] and promotes tumor progression/metastasis [16] through regulation of cytokines, adhesion molecules, metalloproteinases, and proangiogenic activities [71, 72]. Leukocyte infiltration includes B- and T-lymphocytes and TAM also produce TNF which modulate, tumor growth [16, 73, 74]. Indeed, TNF-308 G/G genotypes are significantly higher in patients with RCC compared with healthy controls [75]. Rarely detected in normal TECs, ccRCC, TNF, and TNFR2 protein and mRNA are induced in malignant TECs and infiltrating leukocytes, and TNFR2 increase correlates with malignant/tumor grade [16]. Similarly, increased plasma levels of soluble TNFR2 are detected in patients with metastatic RCC and correlate with malignant grade [76]. 

**Figure 3:**
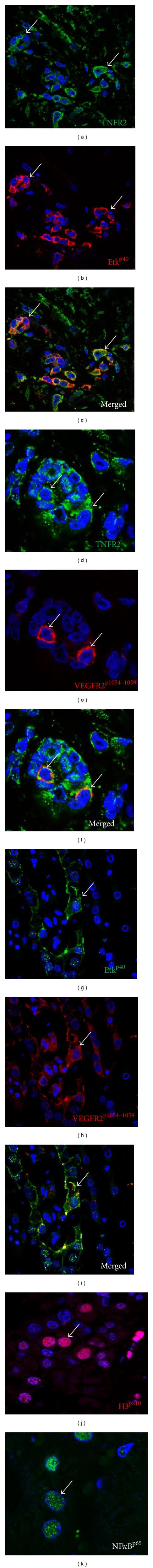
Tumour necrosis factor receptor-2 (TNFR2) mediated cellular responses in organ cultures of clear cell renal carcinoma (ccRCC). Treatment of cultures with a TNFR2-selective mutein induce coordinated activation and phosphorylation of endothelial/epithelial nonreceptor tyrosine kinase (Etk^p40^) and vascular endothelial growth factor receptor-2 (VEGFR2^p1054–1059^) in malignant tubular epithelial cells (arrows) (a–i), promoting cell cycle entry (j), indicated by nuclei staining for phospho-Histone H3^S10^ (H3^pS10^) and NF*κ*B^p65^ activation (k).

In ccRCC organ cultures, a TNFR2-specific mutein induce TNFR2 mRNA and protein comparable to responses elicited by wild-type TNF, although somewhat higher concentrations of the mutein are required to induce the very same responses. TNFR2 induction in TECs in organ culture results in a reciprocal coordinate activation of Etk, VEGFR2, and NF*κ*B and promotes cell cycle entry (indicated by expression of nuclear phospho-Histone H3^S10^ and ki67) (Figure 3), leading to activation of the PI3K/Akt pathway [47]. ki67-positive staining has been proposed as an independent prognostic factor for RCC [77, 78]. TNF-mediated induction of cell proliferation has also been reported in short-term established RCC cell lines [79]. 

Signals induced by TNFR2 in malignant TECs are distinct from the signals induced by VEGFR2, as in contrast to TNF, VEGF does not induce Etk activation or cell cycle entry [16]. Moreover, in ccRCC organ cultures, the downstream effects of TNFR2 signaling occur at much lower concentrations of TNF compared to TNFR1 signalling, consistent with *in vitro* data [80] whereby TNFR2 contribute to the effects of low concentrations of TNF, possibly serving to capture and pass TNF to the less abundant signalling (TNFR1) receptor. The absolute TNFR2 dependence of cell cycle entry in malignant TECs in ccRCC organ cultures using wild type TNF and a TNFR2-selective mutein (but not TNFR1-selective mutein) clearly supports an active signalling rather than a ligand-passing function of TNFR1 [16]. The finding of TNFR2-dependent induction of TNFR2 expression and transactivation of Etk/VEGFR2 in malignant TECs is important as it implies that TNFR2 may act as an autocrine growth factor in ccRCC. In malignant TECs, TNFR2 may regulate gene induction of antiapoptotic proteins via NF*κ*B activation which may halt cell death. Indeed, lack of pVHL protein confers RCC cell resistance to the cytotoxic effects of TNF, which is restored upon reconstitution of the VHL protein, with a concordant expression of NF*κ*B target anti-apoptotic genes such as A20, c-FLIP, Survivin, c-IAP-1, and cIAP-2, blocking the activities of caspases-8 and -3 [81]. 

Single nucleotide polymorphism (SNP) in the TNFR2 gene can predict outcome in other forms of cancer and autoimmune disease [82–85]. A SNP changing T to G in exon 6 at nucleotide 676 of TNFR2 mRNA that results in an amino acid change in the fourth extracellular cysteine rich domain from methionine (TNFR2^196MET^) to arginine (TNFR2^196Arg^) has been associated with increased risk of chronic inflammatory disorders [82, 84, 86]. Expression of the TNFR2^196Arg^ variant in genetically modified cells cause diminished recruitment of TRAF2 to TNFR2^196Arg^ resulting in impairment of TNFR2-mediated NF-*κ*B activation and TNFR1-induced cell death [86]. However, an association between TNFR2 gene polymorphism and ccRCC has not been reported. We have, however, recently demonstrated TNF-mediated induction of TNFR2 in tumor stem cells in ccRCC organ cultures in association with increased cell cycle entry (RSL; unpublished observations). Stem cells isolated from several tumors have been implicated in malignant cell growth and metastasis. Thus, it is not unreasonable to speculate that TNFR2-induction in tumor stem cells may contribute to population of TNFR2-expressing malignant TECs that may be a crucial role in the growth and progression of ccRCC. Alternatively, TNFR2-expressing malignant TECs may provide signals to initiate differentiation of tumor stem cells, which play a role in tubular morphogenesis [87]. Collectively, data from ccRCC organ cultures strongly suggest that TNFR2 in malignant TECs may sustain tumor growth and progression. Indeed, TNFR2 is essential for angiogenesis in murine lung tumor xenografts, as its blockade lead to tumor regression [88]. Moreover, mapping of TNFR2 to chromosome 1p36 revealed that nonrandom translocations occur near this locus in some hematopoietic malignancies [89] and the 1p36 locus has also been associated with malignant lymphoma, neuroblastoma, glioma, and non-Burkitt's lymphoma [89, 90]. 

**Figure 4:**
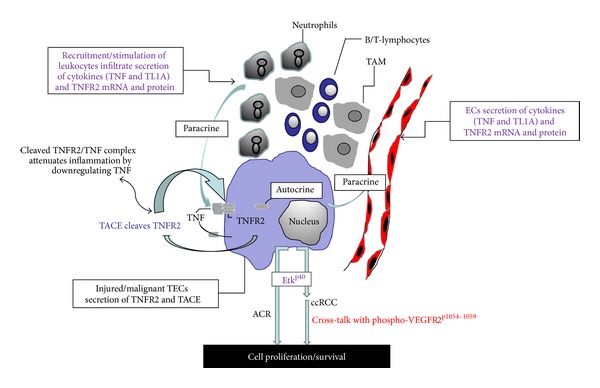
Diagram of postulated events and contribution of the different cell populations in human kidney tissue from biopsies with acute cellular rejection (ACR) and clear cell renal carcinoma (ccRCC). Cytokines (TNF and TL1A) and TNFR2 protein/mRNA secreted by endothelial cells (ECs) and infiltrating leukocytes (neutrophils, tissue-associated macrophages (TAM), and T- and B-lymphocytes) and TNFR2 mRNA and TNF-alpha converting enzyme (TACE) secreted by tubular epithelial cells (TECs) facilitate induction and upregulation of TNFR2 in injured/malignant TECs via autocrine/paracrine mechanisms, promoting activation and phosphorylation of epithelial/endothelial tyrosine kinase at tyrosine 40 (Etk^p40^) and transactivation of vascular endothelial growth factor receptor-2 (VEGFR2^p1054–1059^) leading to cell proliferation/survival. Purple text refers to common effects in ACR and ccRCC and Red text refers to effects in ccRCC.

A model of the contribution of different cell populations to the induction of TNFR2 in TECs in ACR and ccRCC is presented in Figure 4.

## 5. Therapeutic Targeting of TNFR2 in ACR and ccRCC 

Anti-TNF drugs such as infliximab, etanercept, adalimumab, certolizumabpegol, and golimumab have been used for treatment and management of various autoimmune diseases [91–93]; however they fail to show any favorable results in patients with chronic heart failure [94]. So is there a role for anti-TNF drugs in prevention of ACR and RCC? Little is known about anti-TNF therapy in conjunction with antirejection and anticancer in organ transplant recipients. A single-center study evaluating the role of TNF inhibitors in kidney transplantation has been initiated but the results are not yet available [95] and randomized clinical trials are still lacking. Anti-TNF drugs show differential activity and efficacy [96]. For example, infliximab (a chimeric monoclonal antibody) is more effective than etanercept (a p75 TNF receptor/Fc fusion protein) in inhibiting the actions of transmembrane TNF rather than soluble TNF by forming stable complexes [96]. The superiority of transmembrane TNF in activating TNFR2 [97] suggests that under certain conditions, infliximab may be more effective at blocking signalling through TNFR2 than etanercept. A lack of mechanistic understanding of disease contributes to the failure of TNF antagonist. Thus, efforts have been placed in dissecting the individual TNFR signalling responses in an attempt to develop TNFR agonists instead. Selective targeting of TNFR2 in type I diabetes has shown better results than global TNF inhibition [98]. In additional, systemic administration of a TNFR2 agonist to baboons stimulate T cell proliferation without inflammation [99]. The differential expression of TNFRs in ACR and ccRCC provides a therapeutic rationale for developing drugs that target individual receptors rather than targeting global TNF, which might not yield the desired results as this would affect both TNFRs, where TNFR1 signalling is detrimental in ACR while TNFR2 is beneficial, but detrimental in ccRCC. Rather, a much better therapeutical approach would be a selective blockade of TNFR1 in ACR and TNFR2 in ccRCC.

## 6. Conclusion

Experimental data from human organ culture and in *in vitro* systems clearly demonstrate an association of TNFR2 with ACR and ccRCC, suggesting that this receptor may play a crucial role in mediating cellular responses in these disorders. Thus, new therapeutic strategies to either activate TNFR2 signalling in ACR (to facilitate tubular regeneration) or to selectively inhibit TNFR2 signalling in ccRCC (to halt tumor growth/progression) may be a better approach in the treatment of these conditions than global blockade of TNF. Clearly, future clinical studies are warranted to address the prognostic and therapeutic role of TNFR2 in clinical nephrology.
